# Cincinnati College, (Medical Department)

**Published:** 1835

**Authors:** 


### CINCINNATI COLLEGE.

### MANIFESTO.

The Board of Trustees of the Cincinnati College, beg leave to announce to the public, that they have recently instituted and appointed a Medical Faculty. In doing this, they consider it proper to state to their fellow-citizens, the motives and reasons; which have moved them to engage in the enterprize.

At a meeting of the Board, on the 22d of May, it was shown to them on authentic and satisfactory testimony, that the General Assembly of Ohio, at their late session, had appointed a new Board of Trustees for the Medical College of Ohio, with the apparent design of effecting a reorganization of the Faculty of that institution; acknowledged by the Board, and declared by the physicians of the city and state generally, to be in a depressed and unprosperous condition; that after three meetings of the Board, they had been able to proceed no further in the work of reform, than to vacate two of the important professorships; that they had been unable to fill them with new incumbents who would accept; that the country members had returned home, after an adjournment, *sine die*, with the apparent determination to insist on certain resignations in the Faculty, as the condition on which they would again come up to the work of reform; that no resignations were likely to take place, that the College was, therefore, in a reduced and disorganized state, and that one of its ablest professors had expressed the apprehension, that its doors would be closed the ensuing winter. Under these circumstances, the Trustees of the Cincinnati College, could not fail to perceive, and as citizens of Ohio to regret, that the state was in danger of being deprived, at least for the ensuing winter, of the advantages of a good medical institution; and her sons brought under the necessity, in greater numbers than heretofore, of seeking instruction in the distant schools of other states.

To prevent this necessity, was the great object of the Board in organizing a medical department; and as the same condition of the Faculty of the Medical College still exists, after the lapse of several weeks, when more than half the term of the college vacation has expired, the Trustees of this institution, referring to the interests of the profession, of the state and of the city, have much reason to be gratified with what they have done.

They would inform the physicians and students of the West, that they will be able to afford the department an edifice every way calculated for the convenience of both professors and pupils; and that their chartered powers and privileges are the same with those of the University of Pennsylvania, Maryland, or Transylvania, as it respects the creation of a Faculty of any kind, or the granting of correspondent degrees.

In organizing a medical department, the Board, relying on the advice of such of its members as belong to the medical profession, have aimed at making it as full and perfect, in respect to the number of chairs, and the variety of sciences to be taught, as any other medical school in the Union,—and in regard to the character and qualifications of those whom they have appointed as professors, they will say, that the gentlemen have the confidence of the Board, and will, it is not doubted, meet the approbation of the physicians of the West. The titles of the professorships, the names of the professors, the terms of tuition, and all other matters interesting to students, will be set forth in the circular of the Faculty, to which the Trustees beg leave to refer the profession.

By order of the Board.

WM. R. MORRIS, President.

Robt. T. Lytle, Sec. pro tem.

*Cincinnati Ohio, June 27th,* 1835.

---

### CINCINNATI COLLEGE.

MEDICAL DEPARTMENT.

The session will open on the last Monday of October, and continue to the end of February. Full courses of Lectures will be delivered on all the branches of the profession.

Special and Surgical Anatomy,
BY JOSEPH N. McDOWELL, M. D.

General and Pathalogical Anatomy, Physiology and Medical Jurisprudence,
BY SAMUEL D. GROSS, M. D.
(Late of the Medical College of Ohio.)

Surgery,
BY HORATIO G. JAMESON, M. D.
(Late of the Washington Medical College, Baltimore.)

Obstetrics and the Diseases peculiar to Women and Children,
BY LANDON C. RIVES, M. D.
(Late of the state of Virginia.)

CHEMISTRY AND PHARMACY,
BY JAMES B. ROGERS, M. D.
(Late of the Washington Medical College, Baltimore.)

MATERIA MEDICA,
BY JOHN P. HARRISON, M. D.
(Late of Louisville, Kentucky, now of Philadelphia.)

THEORY AND PRACTICE OF MEDICINE,
BY DANIEL DRAKE, M. D.

ADJUNCT PROFESSOR OF CHEMISTRY AND LECTURER ON BOTANY,
JOHN L. RIDDELL, M. A.
(Late of the state of New York.)

Six Lectures will be delivered daily, throughout the session.

Mr. Riddell will teach a private class on Chemistry, by examinations and familiar modes of illustration. His lectures on Botany, strongly recommended by the Trustees and Faculty, but not requisite to graduation, will be delivered in spring and summer.

The price of the ticket of each professor will be fifteen dollars, making an aggregate of one hundred and five — the matriculation fee two dollars — the dissecting ticket ten dollars, and the graduation fee twenty dollars.

Those who intend to graduate must take the tickets of the professors before the 20th of November. No student will be admitted to examination for a degree who is not 21 years of age; of good moral character; has not studied medicine with a private preceptor for three years including the time of his attendance on the lectures; and has not attended two full courses in this College, and taken the dissecting ticket once, or one in some chartered institution, having not less than five professors, and another in this college; but physicians who have been in separate practice four years, may become candidates after attending a single course. Students who attend two courses, and do not wish to graduate at the end of the second term, may continue to attend thereafter as long as they choose, by paying the matriculation fee only.

Clergymen having a pastoral charge, who are desirous of acquiring medical knowledge, but do not intend to change their profession, may attend the lectures gratuitously.

Students and attorneys at law, who wish to avail themselves of the benefits of the lectures on Medical Jurisprudence, can obtain tickets to that course, by paying five dollars each.

Graduates of all other regularly organized and legally authorized medical institutions, will be at liberty to attend the lectures in this institution gratuitously.

The Board of Trustees have furnished the Faculty with ample lecture rooms, in their capacious and central edifice, where those who wish to prosecute practical anatomy, will, under the superintendance of the professor of Special and Surgical Anatomy, be furnished with commodious apartments and all desirable facilities. These apartments will be opened the first of October, to afford students who may desire to engage, personally in operative anatomy, without being interrupted by lectures, the requisite opportunities.

The united cabinets of anatomy — healthy and morbid, human and comparative — of Dr. McDowell and Dr. Gross, will be found extensive, diversified and instructive; and as it is the fixed purpose of the Faculty to render their school eminently anatomical, they intend to adopt such measures as will effect a rapid augmentation of the museum of preparations.

Various courses of lectures by four of the professors, and several gentlemen out of the school, are now delivering and will be continued till the regular session commences in autumn. These lectures are preparatory to the establishment of a permanent summer school, and for this year are gratuitous.

In conclusion the Faculty will add, that every Sunday morning of the three last months of the session, the professor of the Theory and Practice, will deliver a discourse on the social, moral and religious duties of students and medical men; which of course the members of the class will be at liberty to attend or decline, according to their respective tastes.

By order of the Faculty.

DANIEL DRAKE, M. D., Dean.

Medical Faculty of Cincinnati College.

Cincinnati, Ohio, 30th June, 1835.

